# Supplementary figures and images for: An Expanded Landscape of Unusually Short RNAs in 11 Samples from Six Eukaryotic Organisms
Source: Noncoding RNA. 2022 May 19;8(3):34. doi: 10.3390/ncrna8030034 (PMC9149858; doi:10.3390/ncrna8030034)

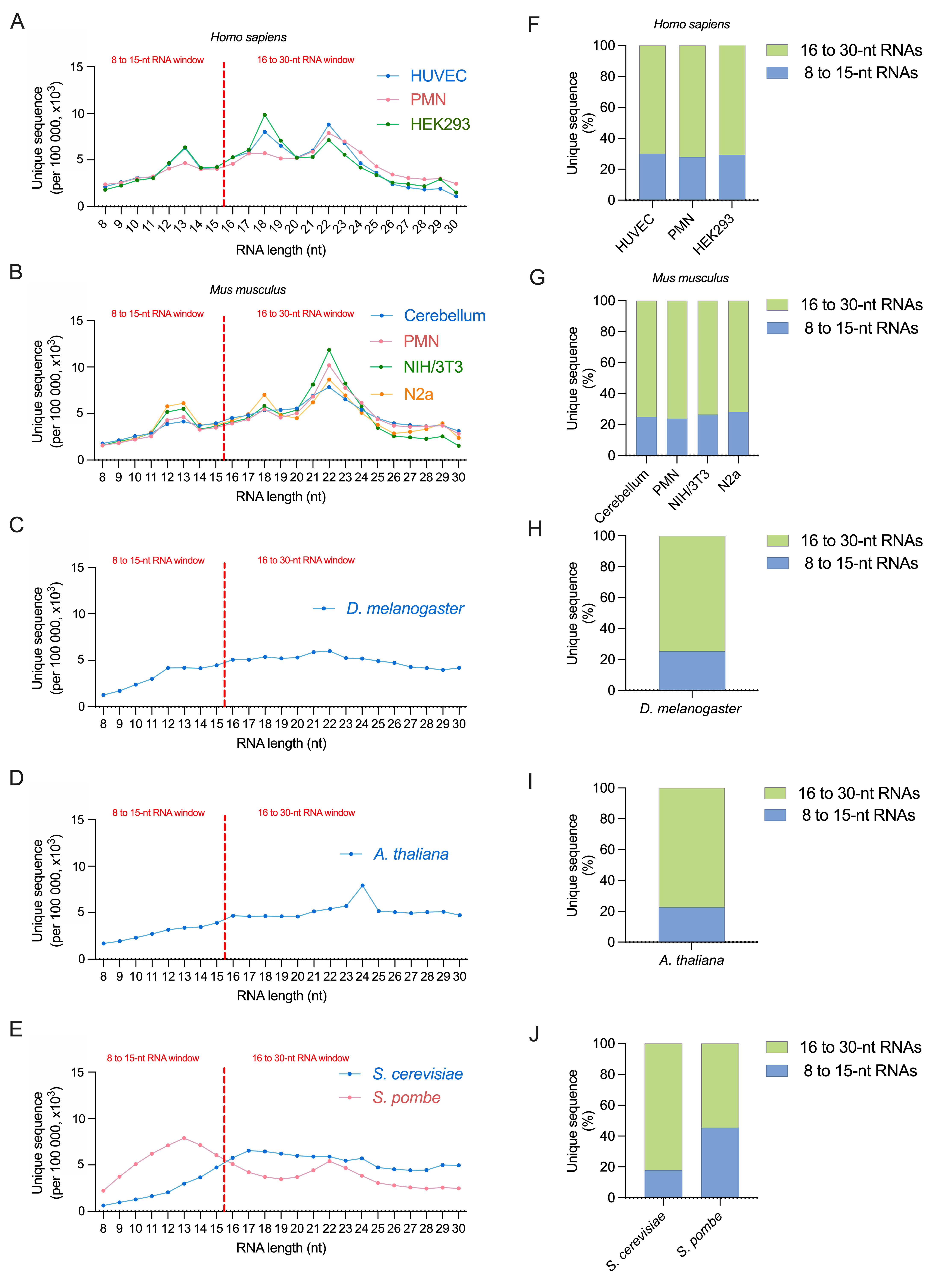

Supplement: Supplementary file 1 [file ncrna-08-00034-s001.zip › 3 Supplementary materials/Supplementary Figure S1.tiff]

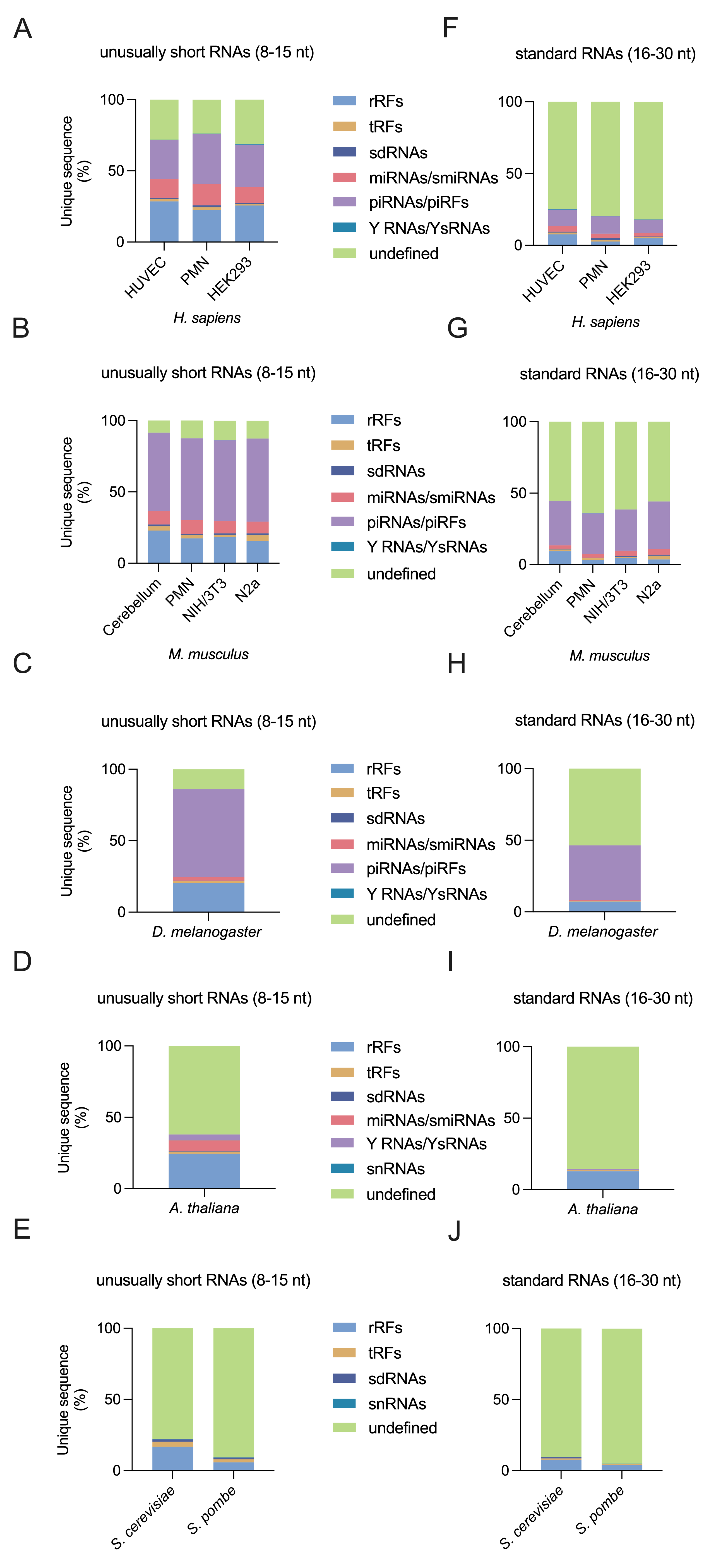

Supplement: Supplementary file 1 [file ncrna-08-00034-s001.zip › 3 Supplementary materials/Supplementary Figure S2.tiff]

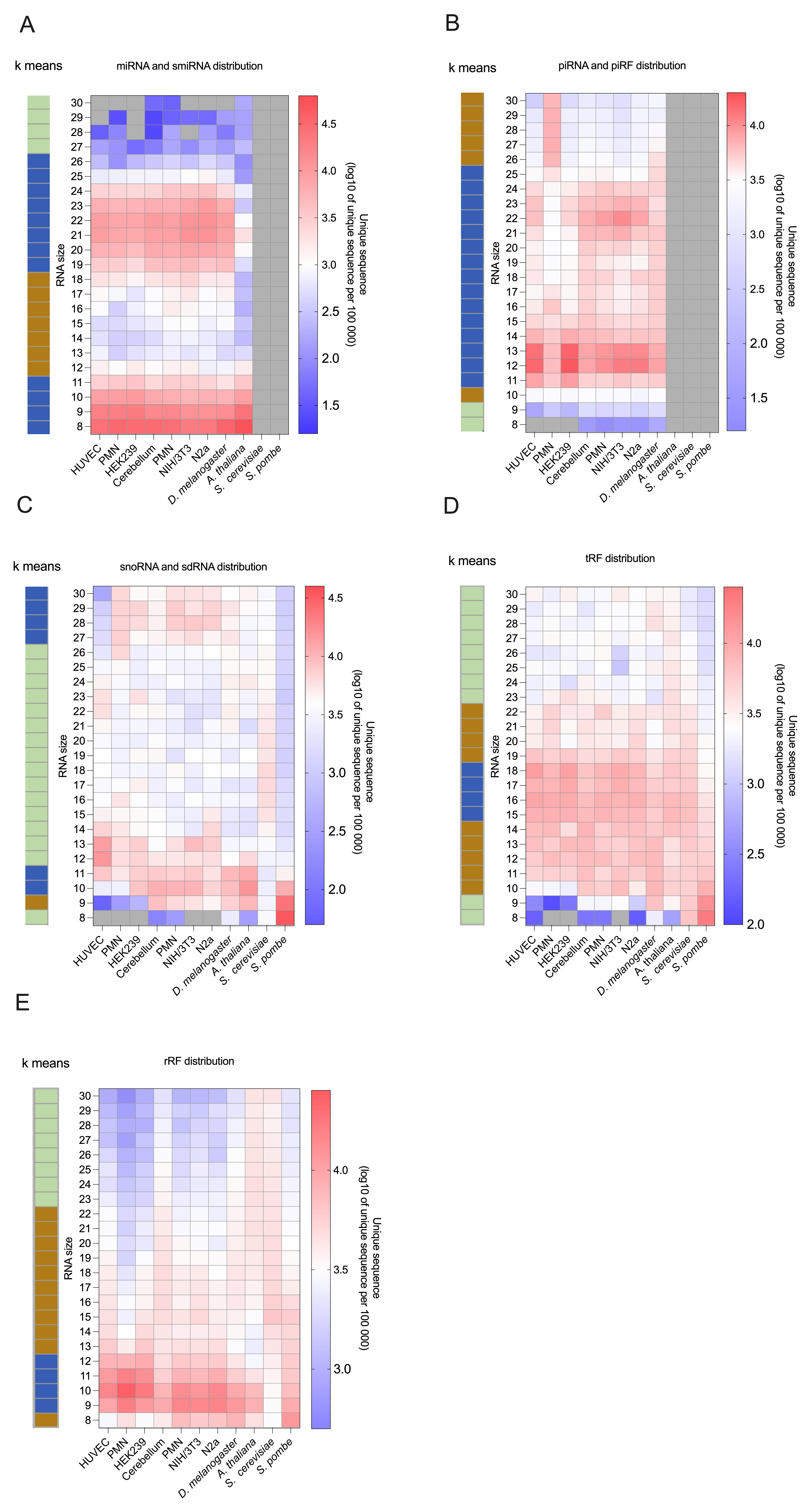

Supplement: Supplementary file 1 [file ncrna-08-00034-s001.zip › 3 Supplementary materials/Supplementary Figure S3.tiff]
